# Inter-professional collaboration in family doctor teams of the Chinese primary care system: a thematic analysis

**DOI:** 10.1186/s12875-025-03129-w

**Published:** 2025-12-05

**Authors:** Hongmin Li, Yushan Ke, Shasha Yuan, Caiyun Zheng, Xin Wang

**Affiliations:** 1 School of Public Health, Jining Medical University, No.133 Hehua Road, Taibaihu district, Jining, Shandong China; 2 School of Public Health, Sun Yat-Sen University, No. 74 Zhongshan 2nd Road, Guangzhou, Guangdong China; 3 Institute of Medical Information & Library, Chinese Academy of Medical Sciences & Peking Union Medical College, Beijing, China; 4 Health Commission of Chengdu, Chengdu, Sichuan China

**Keywords:** Family doctor team, Inter-professional collaboration, Primary care

## Abstract

**Introduction:**

Integrated primary care is advocated worldwide to address the increasing health needs of patients. Inter-professional collaboration (IPC) among family doctor teams (FDTs) aim to provide integrated primary care in China. This study explored how healthcare professionals in FDTs perceive and practice IPC.

**Methods:**

We conducted face-to-face semi-structured interviews with 43 participants from six FDTs, including general practitioners (GPs), nurses, and public health physicians. The interviews were recorded and transcribed, and the transcripts were thematically analyzed.

**Results:**

The analysis revealed three themes: notions, key activities, and elements of IPC in FDTs. Although the concept of IPC varied among interviewees, there were a few commonalities. There was IPC in the delivery of basic medical services (daytime outpatient services and knowledge exchange) and public health services (signing contracts with patients, chronic disease management, health examinations for the elderly, and electronic information entry). The seven elements of IPC include shared goals and vision, communication, leadership, familiarization and trust, time and space for interaction, formalization tools, and incentive mechanisms.

**Conclusions:**

IPC in FDTs in Chinese primary health settings is in the stage of development. We recommend inter-professional competence education for all professionals, adequate interactive space and human resources, supporting cultural environment in primary health institutions (PHIs), and a collaboration-oriented incentive system for FDTs in China.

**Supplementary Information:**

The online version contains supplementary material available at 10.1186/s12875-025-03129-w.

## Introduction

The epidemic of chronic conditions and multi-morbidity is a growing challenge for healthcare systems, especially primary care systems, both in China and globally [1]. Inter-professional collaboration (IPC) among healthcare providers is advocated worldwide to address the increasingly complex health needs of patients with chronic conditions and provide integrated care. IPC is defined as ‘two or more individuals from different backgrounds with complementary skills interacting to create a shared understanding that none had previously possessed or could have come to on their own’ [2]. There is growing evidence that IPC can not only improve care continuity and coordination, patients’ experiences, and health outcomes, but also increase the retention and job satisfaction of healthcare providers [3–5].

Inter-professional teamwork—a model of IPC—is a dynamic process involving two or more healthcare professionals with complementary backgrounds and skills, sharing common goals and exercising concerted physical and mental efforts to assess, plan, perform, and evaluate patient care [6]. Inter-professional teamwork in primary healthcare has been implemented in many developed countries; for example, the integrated neighbourhoods in the UK [7], the Patient-centered Medical Home (PCMH) in the USA [8], the Inter-professional Team in the Netherlands [9], and the Family Health Team in Canada [10]. In the above models, the delivery of continuous and coordinated primary healthcare is based on inter-professional teams. As demonstrated by Reeves et al. [11, 12], team members from different professions collaborating in a cohesive and coordinated manner significantly impact the quality of primary care. D’Amour developed a theoretical framework for IPC—the Four-Dimensional Model of Collaboration—conceptualizing the collaborative process with four dimensions and 10 indicators: shared goals and visions (client-centred orientation vs. other allegiances), internalization (mutual acquaintance, trust), formalization (formalization tools, information exchange), and governance (centrality, leadership, support for innovation, connectivity) [13]. The framework for inter-professional collaboration constructed by Reeves identified the factors affecting IPC, including team composition, time and space, information technology, and organization support, and divided them into four domains: relational, processual, organizational, and contextual domains [14]. Other studies have revealed professionals’ attitudes towards IPC [15], developed assessment tools [16, 17] and evaluated its impact (team environment, clinical outcome, healthcare use, and patient experience) on healthcare teams and patients [18, 19].

Family Doctor Team (FDT) is one such inter-professional team model implemented throughout China to promote professional integration and achieve continuous and coordinated integrated primary care. In China, primary care is mainly provided by community health centers and township hospitals. It serves as the first point of contact for common medical services and basic public health services [20]. FDTs are central to delivering accessible and continuous primary care in the grassroots healthcare networks [21]. In 2016, seven Central government departments jointly issued the *Guidelines on Promoting Contracted Family Doctor Services* [22], regulating primary health institutions (PHIs) to establish FDTs and provide contracted inter-professional care for residents. Generally, an FDT comprises a general practitioner (GP), a nurse, a public health physician, and other healthcare professionals (specialists, traditional Chinese medicine physicians, and pharmacists), if required. Local residents and FDTs signed voluntary contracts, with a period of 1–3 years. These FDTs provide public health services according to the nationwide Equalization of Essential Public Health Service (EEPHS) programme [23], but the other services may vary, depending on the health demands of residents. In related national policies, there are three sources of the contract service fees: the medical insurance funds, Central and local governments, and out-of-pocket fees for contracted residents. However, there is no policy regarding the specific proportion of the fees from the above three sources. Therefore, the amount and proportion of contract service fees significantly vary among provinces. Furthermore, in principle, no less than 70% of the contracted service fees is used for the salary of the FDT members and the contracted service fees is paid after the task performance assessment [24]. By 2021, more than 420,000 FDTs were established, encompassing almost all the cities and counties in China [25].

Although FDTs have rapidly developed over recent years, to the best of our knowledge, only five Chinese studies have targeted IPC in FDTs. Two studies explored IPC prerequisites through qualitative research, encompassing team configuration, service division and information-sharing platforms [26, 27], while three others evaluated the impact of GP-nurse collaboration on managing gestational diabetes and hypertension [28–30]. The existing research fails to address whether and how these prerequisites lead to establishment of IPC within FDTs, which is the critical link between prerequisites and effectiveness. It’s important to understand IPC from the perspectives of FDT members. However, existing research predominantly examines IPC prerequisites from the perspective of institutional managers and evaluates its outcomes from the perspective of patients with chronic diseases, overlooking the viewpoint of healthcare professionals themselves. To address the gap, this study explores the experience of IPC among healthcare professionals in China’s FDTs within the context of integrated care delivery.

## Methods

### Study setting and participants

This qualitative study was conducted in the Huangpu District, Guangzhou City, in August and September 2022, after receiving approval from the Ethical Committee of Public Health, SUN Yat-Sen University. The data were sampled in two steps. First, five community health centres (CHCs) in urban areas and a township health centre (THC) in rural areas were purposely selected from among 17 PHIs. One CHC is a private PHI, while the others are public PHIs. Next, one FDT was randomly selected from each institution. All the members of the six teams were invited to participate in the study. The exclusion criteria were as follows: unable to communicate or not keen to participate. Informed consent was obtained from all participants. The team size was determined based on the principle of data saturation.

### Data collection

Data were collected through face-to-face, semi-structured, and in-depth interviews. The interview guidelines developed based on literature review were validated in the pilot study (Additional file 1). The research team comprised three doctoral-level principal investigators (LHM, WX, YSS) and was assisted by master’s students in public health (ZCY, KYS). All interviewers were female, and all members were trained in qualitative research and ethics. Following an introduction from the head of PHI, the researchers invited team members to participate in the study through face-to-face communication. Upon their consent, the researchers explained the purpose of the study and the interview outline (Additional file 2). Interviews were conducted with all selected team members during working hours in an isolated meeting room at the institution, thereby preserving participants’ personal time. The sessions were confined to the interviewer and participants, with a research assistant present solely without engaging in any interaction. All interviews were conducted with audio recording upon obtaining participant consent. Field notes were taken during interviews by the research assistant and refined within 24 h.

A total of 43 participants were interviewed, with an average interview duration of 30 min. Furthermore, we collected documents on performance appraisal schemes and the division of responsibilities in FDTs as supplementary materials.

### Data analysis

The recordings were transcribed verbatim by one researcher (CZ) and reviewed for accuracy by another (XW). The transcripts were imported into the MaxQda software for thematic analysis based on Braun and Clarke [31]. First, data familiarization was achieved based on the repetitive reading of all transcripts. Second, the relevant data were organized into meaningful codes. Third, the codes were classified into potential themes related to the IPC in FDTs. Fourth, themes were reviewed by reading all the codes and the entire data-set to confirm thematic validity. Fifth, themes were defined and named. Sixth, a report was written. The interviews, transcriptions, translations, and thematic analyses were performed by three principal investigators (XW, HL, CZ). any disagreement regarding coding, classification, or themes during the analysis were resolved through team discussion to reach a consensus.We followed the Consolidated criteria for reporting qualitative research (COREQ) [32]. Selected segments of the transcripts were italicize and embedded in a subsequent report.

## Results

**Fig. 1:**
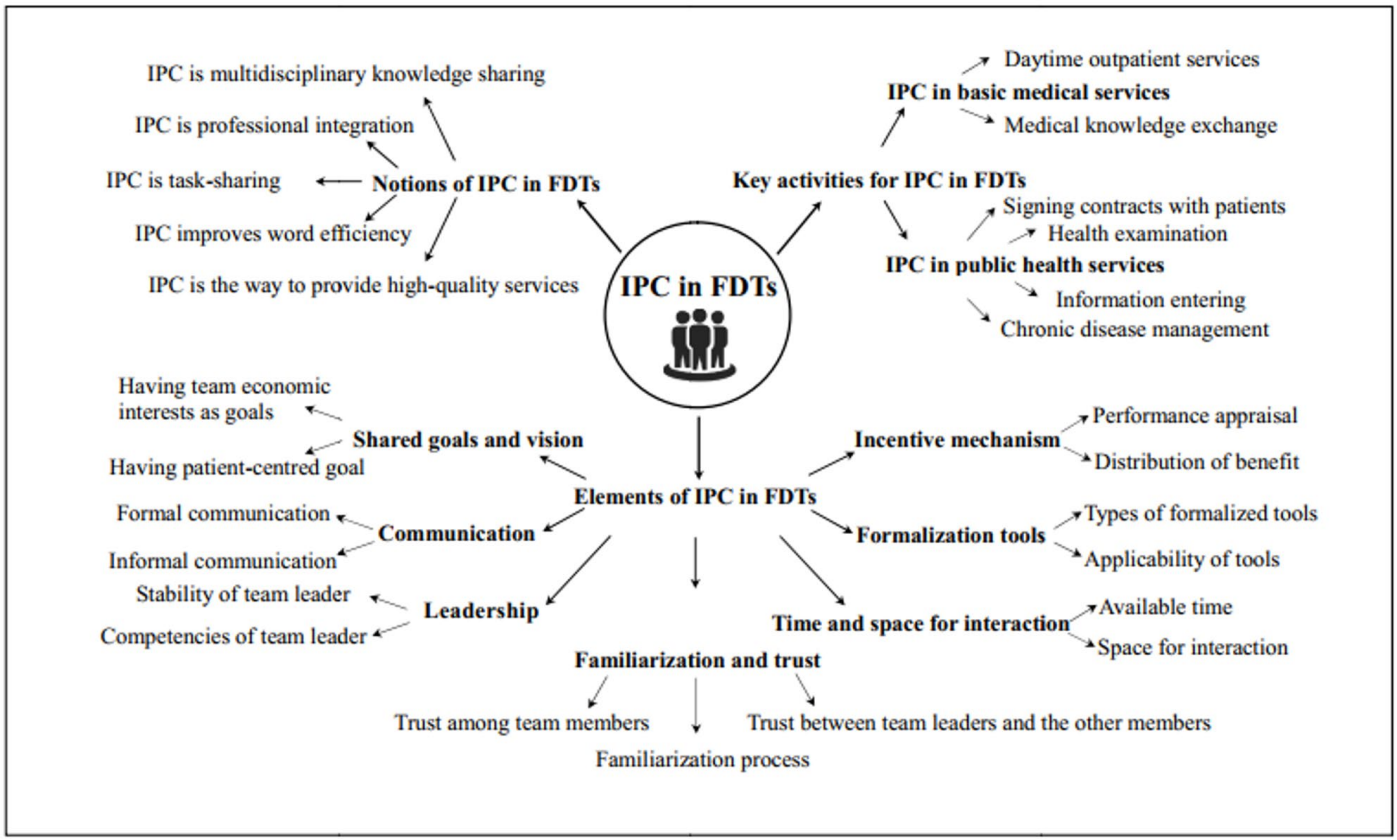
Inter-professional collaboration in family doctor teams

The participants’ characteristics are listed in Table 1. After reading the transcripts, 243 codes related to the IPC in FDTs were generated. As shown in Fig. 1, the codes were categorised under three main themes: (1) notions of IPC in FDTs, (2) key activities for IPC in FDTs, and (3) elements of IPC in FDTs. The themes and sub-themes are shown in Fig. 1. Data saturation was achieved through a total of five teams.

**Table 1:** Characteristics of FDT participants

Team	Type of institution	Type of region	No. of team members	Team composition (No.)	Establishment time	Target population	No. of contracted residents^*^
FDT1	public	urban	4	GP (1), traditional Chinese medicine physician (1), nurse (1), health manager (1)	2017	hypertensive population	3634
FDT2	public	urban	9	GP (3), dentist (1), nurse (3), pharmacist (1), public health physician (1)	2018	general population	400
FDT3	public	urban	5	GP (2), public health physician (1), Nurse (1), GP assistant (1)	2018	general population	819
FDT4	public	rural	13	GP (2), doctor in village clinics (2), nurse (3), pharmacist (1), administrator (3), public health physician (1), traditional Chinese medicine physician (1)	2018	general population	4782
FDT5	public	urban	6	GP (1), public health physician (1) traditional Chinese medicine physician (1), nurse (1), clinical lab technicians (1), GP assistant (1)	2018	general population	450
FDT6	private	urban	6	GP (1), public health physician (1), nurse (1), traditional Chinese medicine physician (1), gynaecologist (1), rehabilitation therapist (1)	2015	general population	2000

^*^Generally, the number of contracted residents for an FDT in Guangzhou is regulated to be less than 2,000. However, the numbers of FDT1 and FDT3 were bigger than 2,000. The target population for FDT1 was the hypertensive population within the service scope of the institution. FDT3 is conducted in rural areas that lack human resources for health, and there are many team members

### Notion of IPC in FDTs

Almost all interviewees reported experiencing IPC, but they differed in their focus when describing its core meaning, such as multidisciplinary knowledge sharing, improvement of work efficiency, meeting demands of patients, task sharing, etc. Although the concept of IPC is understood differently among the interviewees, there are a few commonalities.

‘*IPC implies that members with varied disciplinary knowledge collaborate to address patients’ problems that are difficult to solve by a single professional.’* (FDT4, public health physician). *‘I believe that IPC refers to the mutual assistance of various professionals within a team*,* collaborating to deliver high-quality healthcare services to residents.’* (FDT1, GP). *‘IPC is the integration of multi-disciplinary expertise and skills of team members to better manage patients’ health.’* (FDT2, traditional Chinese medicine physician).

However, a few members believe that IPC implies task sharing.

*‘IPC is about getting the team’s work done better and faster. In a team*,* we have several healthcare professionals to provide care and share tasks.’* (FDT4, doctor in village clinics). *‘The purpose of collaboration is to identify suitable individuals to complete corresponding tasks. In this manner*,* we could improve work efficiency.’* (FDT3, GP assistant).

### Key activities of IPC in FDTs

#### IPC in delivery of basic medical services

The study reveals that collaboration between doctors and nurses constitutes the primary form of IPC in basic medical services provision, with expansion to other professions only in exceptional cases.

Basic medical services were usually provided by doctors (GPs, specialists, traditional Chinese medicine physicians, etc.) in collaboration with nurses. The doctors mainly provided daytime outpatient services to diagnose and treat diseases for residents. The nurses assumed the charge of normal nursing care and assisted the GPs in providing medical services, such as measuring blood pressure before a visit and scheduling a fresh appointment. The GPs, accompanied by the team nurses, visited the residences of patients with mobility problems.

However, when the GPs or doctors in village clinics were unable to visit the patients, they sought help from their team members.

*‘Sometimes patients seek consultation regarding drugs. When I am not sure*,* I ask the team pharmacist for help.’* (FDT2, GP). *‘Once*,* a patient’s blood pressure was too high for me to control*,* and I changed a drug based on the team GP’s suggestion.’* (FDT4, doctor in village clinics).

#### IPC in delivery of public health services

IPC in public health services is characterized by task-oriented, hierarchical teamwork centered on GPs, with under-utilization of non-physician professionals.

IPC in public health services occurs during the process of signing contracts with patients, chronic disease management (for patients with diabetes and hypertension), health examinations for the elderly, and entering information collected for all the 14 public health services into electronic information systems.

The contract-signing process is completed by doctors (GP, specialists, traditional Chinese medicine physicians, etc.) in collaboration with an assistant (nurses, health managers, or GP assistants).

*‘My work involves collecting health information of the newly contracted patients*,* assess their health needs*,* and update the information system so that the GP scan rapidly ascertain patients’ condition and develop care plans.’* (FDT1, health manager).

When introducing contract service packages, doctors usually determine the package based on patients’ needs, without considering the opinions of other members.

*‘After communicating with the patient*,* I ask the assistant to read the contract agreement to the patient*,* and then ask them to join our team’s WeChat group.’* (FDT4, GP).

Chronic disease management involves health education, four follow-up visits, and one health examination per year. Although GPs are responsible for follow-up visits in the outpatient clinic independently, they have to provide health education and conduct examinations in collaboration with other members. In particular, the annual health examination of elderly patients over 60 years of age and patients with chronic diseases is usually the collective action of all members.

*‘First*,* we discuss the task arrangement*,* material preparation*,* and work duration. Thereafter*,* the nurse usually distributes the health checklist to patients and guides them to fill out the basic information. The GPs and doctors in village clinics conduct physical examination*,* B-ultrasound examination*,* electrocardiogram*,* etc. assisted by me and other members. Finally*,* we enter the collected data into the information system together.’* (FDT4, public health physician). *‘I distribute various electronic information entry tasks to team members based on their willingness and workload.’* (FDT5, GP).

Therefore, most FDTs members enter information related to all the 14 public health services into the electronic information system by task-sharing. However, members usually collaborate to complete these tasks.

*‘Sometimes they are extremely busy*,* and I help them with their tasks when I am free because we are a team. We all help each other.’* (FDT1, traditional Chinese medicine physician).

It is noteworthy that most public health physicians complained that they performed non-professional activities and could not contribute their professional knowledge or skills in the collaboration.

*‘I am not clear about my role in the team and how to perform it. As the current policy requires GPs to manage patients with chronic diseases*,* my daily routine is to electronically record the information of residents or help with health examinations. It is extremely unfulfilling.’* (FDT3, public health physician).

Respondents of FDT4 even suggested that GPs were not accustomed to or were unwilling to distribute key tasks to them.

#### Elements of IPC in FDTs

The interviews highlighted seven elements of IPC in FDTs: shared goals and vision, communication, leadership, familiarization and trust, time and space for interaction, formalization tools, and incentive mechanisms.

#### Shared goals and vision

Most interviewees recognize that a clearly defined shared goal is critical to effective IPC, whereas its absence undermines teamwork and role clarity.

Having shared goals and vision was identified as the paramount element of IPC within teams.

*‘I think*,* first*,* we should have a common and explicit goal*,* so that we remain motivated and enthused.’* (FDT6, GP).

A few teams lacked common goals and vision, which had a significant negative impact on members.

‘*To be honest*,* (the lack of shared goals) confused me regarding what I or others could contribute*,* or what is the direction of our team. I had no sense of collaboration to achieve the goals.’* (FDT5, nurse).

Teams with shared goals mainly had two different goals. Certain teams regarded economic interests as a common goal. *‘The goal is to contract more patients; we intend to obtain the first place in the institution.’* (FDT2, GP). The other teams shared patient-centric goals. *‘We intend to be a good FDT*,* providing high-quality health management for patients and meeting their needs.’* (FDT6, nurse). However, a few respondents indicated that it was difficult to achieve this goal with limited human resources and time.

#### Communication

Respondents recognize that effective interprofessional collaboration relies on a combination of formal and informal communication, with informal channels being used more frequently for daily problem-solving.

Constant and active communication helps members understand each other’s personalities, responsibilities, and contributions and establishes mutual trust and respect.

*‘Only mutual communication can reveal what team members think and facilitate agreements.’* (FDT6, public health physician).

Team members in FDTs mutually interact via formal (seminars and meetings) and informal (luncheons, afternoon parties, and chats on social media) communication channels. Almost all participants indicated that in formal communication occurs more frequently than formal communication, and that they rarely have regular meetings. However, they have meetings or huddles with team members as and when required.

*‘Our nurse and I could meet at any time and communicate directly*,* which not only saves time but also solves problems. Honestly*,* formal meetings are rarely held because of time constraints.’* (FDT6, traditional Chinese medicine physician). *‘We often discuss regarding patients’ care over lunch.’* (FDT3, nurse).

However, a few respondents also suggested the necessity of formal communication.

*‘We should meet regularly to summaries the progress of each other’s work and share useful experiences*,* information*,* etc.’* (FDT2, pharmacist).

Moreover, although online communication is convenient, most members prefer face-to-face communication to avoid misunderstandings.

#### Leadership

GPs were responsible for performance evaluation of members and managing issues in all six teams. Most interviewees view leadership as a key component of effective IPC and expressed strong support for the prevailing GP-dominated leadership structure.

*‘In addition to my own work*,* I am responsible for task allocation*,* management and assessment of team members*,* and organizing team training and other activities.*’ (FDT3, GP).

Except for the GP in FDT3, who was selected based on competency assessment, other GPs were directly appointed by the institution. Interestingly, a few team leaders adopted effective approaches to ensure the efficient operation of the team, such as assigning experienced members to mentor novice members and organizing team members to mutually verify work quality.

Under the effective management of team leaders, members can explicitly define their responsibilities and roles and master inter-professional knowledge and skills. Team leaders’ contributions toward creating a positive, trusting, and open team environment was highlighted by members of six teams. Meanwhile, many interviewees proposed the required competencies of team leaders.

*‘For the team*,* they are like a rock. When I have any suggestion or opinion*,* I share with them. They should remain fixed persons*,* display organizational ability*,* have effective communication skills*,* possess a sense of responsibility*,* and play a leading role.’* (FDT5, public health physician).

#### Familiarization and trust

Many interviewees classified familiarization and trust as the underlying preconditions of IPC. Joint training, inter-professional care delivery, and daily communication promote familiarization among these teams. Several respondents commented on the significance of intimate mentor-ship resulting from mutual familiarization.

*‘The intimate relationship established after acquiring familiarity with the GP has improved my professional abilities and skills…and helped me gain greater trust of patients.’* (FDT4, doctor in a village clinic).

Two types of trust within FDTs have been reported: trust among team members and trust between team leaders and other members. The latter is more difficult to construct than the former.

‘*I think they (members) still require time to adapt to their roles in the teams*,* and I also require time to build my trust in them.’* (FDT5, GP).

The interviews showed that trust within a team is conditional and mainly based on members’ abilities (knowledge and skills), attitudes, and experiences.

*‘We depend on her (GP) a lot and follow her advice because she has rich work experience and is a nice human being.’* (FDT1, nurse).

#### Time and space for interaction

Availability of time and space for interaction among team members as also been proposed as an element of IPC.

The members of a FDT were employees of the same PHC facilitates, so all team members shared space, except two doctors in the village clinics of FTD4. FDT1 even arranged all team members in the same office. The interviewees commented that the space for interaction in FDTs considerably promoted opportunities for face-to-face communication and mutual acquaintanceship, and reduced unnecessary misunderstandings.

*‘As we are all in the same office*,* we can communicate at any time*,* which is a good think. Everything is settled in person.’* (FDT1, health manager). *‘When there are problems*,* I go upstairs directly to discuss with them before the situation deteriorates.’* (FDT3, GP).

Some interviewees mentioned about conflicting schedules and heavy workloads limit opportunities for interaction.

*‘I have been extremely busy since the COVID-19 pandemic. I serve at a fever clinic in the morning*,* conduct nucleic acid tests at a quarantine hotel during 12:00–15:00*,* and then return to the emergency department. Our heavy workload negates the possibility of performing the shared tasks.’* (FDT4, GP). This varied and busy schedule of team members significantly affects interactions in FDTs, hindering IPC. *‘One of us goes to conduct nucleic acid tests for residents every day. I have not met them since this Monday.’* (FDT5, public health physician).

#### Formalization tools

Formalization of IPC in the interviewed teams rely on standard national guidelines, integrated information systems, and team-level agreements on performance and responsibilities.

First, all teams mainly followed the guidelines of the EEPHS programme formulated by the national government. The guideline defined the care packages of the 14 public health services for the target populations, including the standardized care pathways, scheduled follow-up, and structured management plan. Second, information sharing was based on information systems uniformly developed by the districts. The assistant professionals (nurses, health managers, and GP assistants) usually updated the information system, such as health examination information and follow-up records, to ensure that all members can obtain complete health information of residents for high-quality health management. Third, a few teams developed performance distribution plans that defined the distribution standard and proportion of the contract service fees. The plans were proposed by the GP and concluded after consensus of all members. The formalization of collaborative relationship in a few teams was based on the division of responsibility agreements, however, these agreements are drawn up by the team leaders without united standards. Furthermore, several respondents highlighted that the division of responsibilities agreement was not currently applicable.

*‘Before the COVID-19 pandemic*,* our division of responsibilities agreement was meaningful; but now*,* we are also responsible for prevention and control of COVID-19. Sometimes*,* it is really impossible to follow the agreement.’* (FDT1, nurse).

#### Incentive mechanism

Transparent and reasonable performance appraisals and distribution of benefits were identified as elements of IPC by all teams. The design of appraisal and benefit-distribution, particularly the metrics for evaluation and the method of fee distribution, directly influence members’ motivation for IPC.

*‘If the appraisal system is transparent and an incentive mechanism is in place*,* they will be significantly motivated to work and collaborate. In my opinion*,* that is the only way for me to motivate them.’* (FDT4, GP).

The performance appraisal of FDTs involved the assessment of each team by PHIs and each member by the respective teams. The appraisal by many PHIs was mainly based on the number of registered residents and services provided, which induced team members to blindly pursue the number of services and pay scant attention to the quality of services. *‘Now we are attempting to register more residents. Our team was asked to contract 500 patients this year. The maximum is 2000.’* (FDT5, public health physician). To ensure service quality, the appraisal by a few PHIs involved related strategies. *‘The appraisal assesses the quality of care*,* such as blood pressure control and patient satisfaction.’* (FDT6, GP). Thereafter, the team leaders evaluated each member based on their workload.

Team leaders generally have the power to distribute contracted service fees within the teams. The distribution method and frequency of payments are key components of incentive mechanisms. Most teams resorted to proportional allocation; the remaining resorted to equal allocation. In the allocation, the proportion of team leaders was higher than that of other members, and the proportion of GPs in a few teams was 5–20% more than that of other members. This resulted in conflicts within the teams. *‘The nurses complained that although the doctors performed less work*,* they were paid more.’* (FDT5, GP). Conversely, equal allocation in a few teams reduced leaders’ motivation. *‘If I do not sign a contract with patients*,* there will be no benefits for anyone.’* (FDT2, GP). A majority of the respondents preferred to allocate service fees with minor differences among members. Moreover, quarterly, rather than annual, distribution of benefits provided greater motivation to the members. It is noteworthy that for a few interviewees, the service fees had little impact on their motivation for collaboration.

*‘Providing service significantly increase my workload*,* but the contracting service fee constitutes only a minor part of my performance-based salary*,* which makes little sense.’* (FDT5, GP).

## Discussion

### Main findings

This pioneering study explores team-based practices in Chinese primary care from the IPC perspective, revealing three thematic areas: notions, key activities, and elements. Overall, there is a gap between participants’ understanding of IPC and the definitions by the World Health Organization (WHO) and European Union (EU) [4, 33]. Although the reported elements correspond to D’Amour model [13], cognitively, personnel primarily view IPC as task-sharing, with scant recognition of shared goals or inter-professional learning. According to the forms and stages of IPC by WHO, EU and D’Amour model, the IPC in FDTs of Chinese primary care systems has not yet been fully established. It remains largely a task-based-orientation care delivery chain, lacking a formal vision, mission, and process of IPC.

A comprehensive and accurate perception of IPC is contingent upon a supportive cultural environment. The lack of a common IPC culture in China’s primary health system is a constant barrier to IPC. Traditional hierarchies among disciplines exist in our country, as they do elsewhere [34, 35], especially between doctors and other professionals. Moreover, this asymmetry and inequality among professionals is variously reflected-for example, the income growth rate of GPs is significantly higher than that of other healthcare professionals in the same institution [36]. The traditional hierarchical power structure controlled by doctors facilitates efficiency [37]. However, in inter-professional teams, doctors are always in the dominant position, which inevitably leads to a power imbalance among healthcare professionals, resulting in non-inclusive, non-shared decision-making processes and limited co-ordination. Equality among healthcare professionals is an essential characteristic of collaborative practice [38]. Unfortunately, many researchers have highlighted the difficulty of achieving hierarchy and asymmetry among professions. Anum et al. discussed the challenges in transforming from the traditional doctor-led model to the team model in primary care setting [39]. In China, this is especially difficult as doctors possess a monopoly on prescribing, a deeply entrenched professional culture, and disproportionately high patient trust. The complete absence of any mention of equality by interviewees in this study points to an absence of awareness among FDT members regarding the necessity of power sharing and truly collaborative practice.

The absence of inter-professional learning in the notion of IPC reveals a critical gap in education and training. It is evident that healthcare professionals have to effectively collaborate. However, as stated in the Lancet Commission, professionals lack the required competencies for effective teamwork [40]. Similarly, throughout their education and internship, Chinese medical students are immersed in the values and basic theories inherent in their own profession, with little knowledge of the practices, expertise, skills, and values of professionals in other disciplines.As one mixed-method study showed, knowing each other’s expertise is necessary for effective collaboration among team members [41]. However, the existing education system tends to foster and support individualism, specialization, and domain thinking rather than collaborative practices. Several studies have identified this domain thinking as a barrier to the process of shared care plan development. Team members protect the scope and practice of their particular specialty in terms of identity autonomy and accountability [42]. Additionally, GPs in FDTs lack the necessary leadership awareness and ability. Actually, as Smith concluded, effective inter-professional team leadership requires a unique mix of competences supporting innovation and improvement [43].

The incentive mechanism is an additional factor identified in this study that falls beyond the scope of the D’Amour model. An unreasonable incentives mechanism for FDTs hindered IPC and encouraged task orientation in teams. In 2016, to achieve a wide coverage of FDT contracting service, the government set an extremely high contracting rate as the goal. Therefore, PHIs developed the performance appraisal scheme focusing on the number of contracted residents rather than comprehension or quality of care provided. Previous studies emphasize that shared people-centric goals and vision can generate valuable collaborative actions [44]. However, in such contexts, professionals tend to pursue more contracted patients and more contract service fees, undermining the motivation to collaborate with other members and provide people-centric integrated care to patients. Moreover, the quantity-oriented performance appraisal scheme leads to low work efficiency and poor care quality [45].

Findings from the elements “Familiarization and trust” and “Time and space for interaction” reveal that inadequate staffing and high turnover rates were the underlying challenge to maintaining consistent IPC. In 2022, the number of GPs per 10,000 people was merely 3.2 in China, and the quality of professionals in PHIs was significantly lower than in hospitals [46]. Additionally, research has identified the frequent turnover of professionals in PHIs [47], which not only hinders the continual availability of qualified professionals, but also directly disrupts interpersonal relations among members [48, 49]. We noted that COVID-19 had aggravated the situation to some extent. As arranged by PHIs, many healthcare professionals were devoted to COVID-19 prevention and control. This hindered the interaction, communication, and collaboration among healthcare professionals in FDTs.

Findings from the element “Time and space for interaction” reveal that, different from countries where GPs practice independently or have team members working in different locations, team members in FDTs in China worked at the same institution. This implies that they often have shared work spaces and fixed discussion places. Co-location of professionals is often cited as a key enabler of collaborative practices [50, 51], which could not only facilitate formal or informal communication, but also reduce the power gap among professionals [43, 50]. Regarding the element “Formalization tools”, the national policies explicitly defined FDTs’ composition, promoted formal partnerships and increased the stability of collaborative practices. Meanwhile, formalization tools have been developed at the national, municipal, institutional, and team levels. Although shared decision-making is not always successful in ensuring agreements at the team level, these tools are useful for sharing responsibilities, dividing responsibilities among members, and standardizing public health service processes [37].

### Implications for policy and practice

To strengthen primary care professionals comprehensive understanding of IPC, the following aspects need to be addressed. On one hand, in-service training should be enhanced with content covering chronic disease co-management and interprofessional communication, with emphasis placed on training effectiveness. On the other hand, in medical education, theoretical and practical courses on IPC should be established to improve students’ awareness and literacy of IPC.

At the practical level, in contexts where systematic IPC practices have not yet been established, the following measures are necessary. First, improve the support system for IPC in terms of space, human resources, funding, and information sharing. For example: creating shared workspace and joint consultation rooms to reduce communication barriers, allocating dedicated funds to support collaborative initiatives, and implementing unified electronic health record systems to ensure service continuity. Second, reform the quantity-oriented performance evaluation and incentive mechanisms by incorporating metrics such as frequency of collaboration and number of joint case management episodes into performance assessments. Third, enhance non-financial incentives for IPC practice to reinforce team willingness to collaborate.

### Strengths and limitations

Using face-to-face semi-structured interviews, we explored the notion, activities, and elements of IPC in FDTs to reveal the status of IPC and the reasons behind it. Recruiting FDTs with variations in institution and geography purposefully ensured the diversity of views and richness of data. However, this study has several limitations. First, we sampled within a city. Given that FDT implementation varies across regions, we should be cautious when generalizing the results of this study for policy and practice. Second, the findings of this study were based on interviews with all members of the six FDTs. Interviews with other stakeholders, patients, and managers of primary care institutions may help interpret IPC in FDTs from various perspectives.

## Conclusion

Currently, IPC in FDTs in Chinese primary health settings is at a collaborative yet developing stage. The elements of IPC described by participants mostly align with the existing frameworks, with limited recognition of the importance of shared goals or inter-professional learning. These findings will inform policymakers regarding IPC promotion and delivery of integrated primary care in China and other developing countries with weak primary care settings. Based on the findings in China, we suggest education and training regarding essential inter-professional competence for all professionals, creation of interaction spaces, increment of human resources, development of appropriate cultural environments in primary care institutions and development of a collaboration-oriented appraisal and incentive system for FDTs.

## Supplementary Information

Supplementary Material 1: Additional file 1: Table S1

Supplementary Material 2: Additional file 2: Interview Guideline

## Data Availability

The data analyzed in the present study are available from the corresponding author (XW) upon reasonable request. To safeguard the identities of study participants, full interview data—including audio files and transcripts—will not be publicly available.
