# Supplementary material for: Inter-professional collaboration in family doctor teams of the Chinese primary care system: a thematic analysis
Source: BMC Prim Care. 2025 Dec 5;27:53. doi: 10.1186/s12875-025-03129-w (PMC12888562; doi:10.1186/s12875-025-03129-w)
Supplement: Supplementary file 1 — Supplementary Material 1: Additional file 1: Table S1 [file 12875_2025_3129_MOESM1_ESM.docx]

**Additional file 1**

Table S1 The characteristic of participants

| Demographic characteristics | N (%) |
| --- | --- |
| Sex |  |
| Male | 7(16.28%) |
| Female | 36(83.72%) |
| Age |  |
| <35 | 10(23.26%) |
| [35-45) | 26(60.47%) |
| ≥45 | 7(16.28%) |
| Education level |  |
| College | 3(6.98%) |
| Undergraduate | 38(88.37%) |
| Graduate | 2(4.65%) |
| Years of working |  |
| <1 | 2(4.65%) |
| [1-5) | 3(6.98%) |
| [6-10) | 12(27.91%) |
| ≥10 | 26(60.47%) |
| Professional |  |
| General practitioner | 10(23.26%) |
| Nurse | 10(23.26%) |
| Public health physician | 5(11.63%) |
| Traditional Chinese medicine physician | 4(9.30%) |
| Specialist | 2(4.65%) |
| Others^#^ | 12(27.91%) |

^#^Others include pharmacist, doctor in village clinics, rehabilitation therapist, general practitioner assistant, administrator, clinical lab technician and health manager.
