# Supplementary material for: Inter-professional collaboration in family doctor teams of the Chinese primary care system: a thematic analysis
Source: BMC Prim Care. 2025 Dec 5;27:53. doi: 10.1186/s12875-025-03129-w (PMC12888562; doi:10.1186/s12875-025-03129-w)
Supplement: Supplementary file 2 — Supplementary Material 2: Additional file 2: Interview Guideline [file 12875_2025_3129_MOESM2_ESM.docx]

**Survey on the Collaboration Level and Influencing Factors of Family Doctor Teams (Inter-professional collaboration)**

Survey Date: ____/__/__

Surveyor: ______

Name of Community Health Center: ______

| **1. Basic Information** | | |
| --- | --- | --- |
| **Team Basic Information** | 1. Total Number of Team Members: ______  2. Team Composition: ______  3. Year of Team Establishment: ______  4. Total Number of Residents Served: ______ | |
| **Individual Basic Information** | 5. Position: □ General Practitioner □ Specialist Physician □ Nurse □ Public Health Physician □ Traditional Chinese Medicine (TCM) Physician □ Others  6. Are you a staff member of this Community Health Center? □ Yes □ No  7. Gender: □ Male □ Female  8. Age: ______ years old  9. Educational Background: □ Secondary Vocational or Below □ Junior College □ Bachelor’s Degree □ Master’s Degree or Above  10. Years of Work Experience: □ < 1 year □ 1–5 years □ 6–10 years □ > 10 years  11. Number of Family Doctor Teams You Are Part of: □ 1 □ 2 □ 3 □ > 3 | |
| **2. Interview Questions** | | |
| **Shared Goals and Vision** | | |
| Shared Goals | | 1. What are the work goals of your family doctor team? Do you agree with these goals?  2. Who proposed the above team goals? (What were the standards and process for setting them?)  3. What impact has the confirmation of team goals had on you or the team? |
| Patient-Centeredness | | 4. What factors affect your service provision to contracted residents? (Hints: Patients’ individual conditions, the institution’s medicines/medical equipment, influence from leaders or colleagues, etc.)  5. Which of these factors is the most critical? |
| **Internalization** | | |
| Familiarization | | 6. Do you have daily work communication with other members of your family doctor team?  a. If yes: Please describe the form/channel, frequency, and content of the communication.  - Which form of communication do you think is most efficient? Which frequency is most appropriate? Why?  - What impact have these communications had on you or the team?  - Do you understand the capabilities, experience, team roles, and division of labor of other team members?  b. If there is no daily communication or communication is ineffective: What are the main reasons?  - Do you have any suggested solutions for these reasons? |
| Trust and Respect | | 7. In daily work, do you trust the professional skills and responsibility-taking ability of other team members? (You may analyze which members you trust/distrust and the reasons one by one.)  8. Do you think other team members have carefully listened to and adopted your opinions? Do they trust your professional skills and responsibility-taking ability? (Please provide examples.)  a. If there is mutual trust: How did your team establish an atmosphere of mutual trust and respect? What factors are conducive to building mutual trust and respect among team members? What impact has this trust and respect had on you or the team?  b. If there is no mutual trust yet: What factors hinder the establishment of mutual trust and respect among team members? Do you have any suggested solutions for these obstacles? |
| **Leadership** | | |
| Policy and Administrative Support | | 9. Are you aware of the policy documents that support and guide the services of family doctor teams?  a. If yes: Please give examples (national/municipal level). What impact do you think these policy documents have had on the services of family doctor teams?  10. Do the leaders (e.g., director) of the Community Health Center support the development of family doctor teams?  a. If yes: Please describe the specific support provided by the leadership of the Community Health Center. What impact has this support had on you or the team? |
| Team Leadership | | 11. Who do you think is the leader of your family doctor team? What are his/her job responsibilities?   1. 12. Is he/she competent as the team leader? Why?   13. What impact has the team leader had on you or the team? |
| Decision-Making Mechanism | | 14. Can you describe how the decisions and plans of your family doctor team are made? What is the formulation process?  15. When your family doctor team encounters problems (e.g., declining service effectiveness or reduced resident satisfaction), is the team capable of solving the problem?  a. If yes: How is the problem solved?  b. If no: What are the reasons? |
| Innovation Support | | 16. Has your family doctor team conducted training or guidance on collaboration/cooperation?  a. If yes: Were these training or guidance useful? What impact have they had on you or the team?  b. If no: Do you think such training or guidance is necessary? What are the reasons?  17. Do you think your family doctor team needs to innovate/adjust its service delivery model, or adjust its staffing or division of labor?  a. If yes: Can you obtain technical support to know how to make such adjustments? If yes, who provides the support?  b. If no: Why? |
| **Standardization** | | |
| Service Norms | | 18. Has your family doctor team formulated documents such as team members’ responsibility division agreements, team work plans, or team service manuals? (To be supported by documentary evidence)  a. If yes: How were these agreements and plans formulated? Do they play a role in standardizing service provision in daily work or practice? What impact have they had on you and the team?  b. If no: Do you think it is necessary to formulate such agreements and plans? What are the reasons? |
| Interconnection of Health Information | | 19. Can members of your family doctor team share patients’ health information (including health records, electronic medical records, examination and test results, etc.) with each other?  a. If yes: Are the channels/systems for this information sharing unobstructed and efficient (e.g., for accessing electronic medical records/health records)? What impact has this had on you or the team?  b. If no: What are the obstacles? |
| **Incentives** | | |
| Performance Appraisal | | 20. Is there performance appraisal based on the family doctor team as a unit?  a. If yes: Please describe the performance appraisal items related to your family doctor team (intra-team appraisal/institutional appraisal). Explain the appraisal content, frequency, and feedback one by one.  - How were the above performance appraisal methods formulated? Did you participate in their formulation?  - Are these performance appraisal methods reasonable? What impact have they had on you or the team?  b. If no: Do you think it is necessary to conduct performance appraisal based on the family doctor team as a unit? What are the reasons? |
| Benefit Distribution | | 21. Is there benefit distribution based on the family doctor team as a unit?  a. If yes: Please describe the benefit distribution method related to your family doctor team (whether it is linked to performance appraisal results, and what incentive measures are provided).  - How was the above benefit distribution method formulated? Did you participate in its formulation?  - Is this benefit distribution method reasonable? What impact has it had on you or the team?  b. If no: Do you think it is necessary to conduct benefit distribution based on the family doctor team as a unit? What are the reasons? |
| 22. Can you talk about the challenges and obstacles encountered in the process of team collaboration? Why do you consider it as a challenge/obstacle? Has it been resolved so far? How was it resolved?  23. What factors do you think are important factors and prerequisites for promoting team collaboration? Why?  24. What suggestions or expectations do you have for the future development of your family doctor team? | | |
